# Uterine Rupture Secondary to Pyomyoma, Leading to Intra-Abdominal Abscesses following an Uncomplicated Vaginal Delivery

**DOI:** 10.1155/2023/3306687

**Published:** 2023-10-04

**Authors:** Rachel Hartman, Olga Colón-Mercado, Valario Johnson, James Baron, Lauren Davis

**Affiliations:** HCA Healthcare/USF Morsani College of Medicine GME Program, Brandon Regional Hospital, Brandon, FL, USA

## Abstract

**Background:**

Pyomyomas are an infrequent complication of uterine fibroids and, in extremely rare cases, the cause of spontaneous uterine rupture. A few documented cases were managed conservatively with oral antibiotics and CT-guided drainage or myomectomy with fertility preserved. However, treatment more frequently involves IV antibiotics and a hysterectomy. *Case Description*. A 31-year-old G2P0111 PPD 7 presented with intra-abdominal abscesses of unknown source. She was treated with broad-spectrum antibiotics, image-guided percutaneous (IR) drainage of the largest abscess, and surgical exploration with debridement. During surgery, she was diagnosed with spontaneous uterine rupture. The uterine defect was successfully repaired, and she was able to be successfully managed with fertility-sparing treatment. The patient ultimately did not require a hysterectomy. The final pathology was consistent with pyomyoma.

**Conclusion:**

In a majority of cases, pyomyoma treatment requires a hysterectomy, and fertility is unable to be preserved. However, conservative management with IV antibiotics, IR drainage, and surgical debridement could be a fertility-preserving approach to the treatment of pyomyomas.

## 1. Introduction

Uterine leiomyomas, or fibroids, are benign monoclonal tumors that develop from the myometrium's smooth muscle cells and fibroblasts. They are very common in reproductive-age women and are quite common during pregnancy [1]. The prevalence of uterine fibroids during pregnancy was reported to be as high as 10.7% when including all races and ethnicities, but prevalence was reported to be substantially higher in Black women (18%) [2].

Uterine fibroids during pregnancy are typically asymptomatic. When they do cause symptoms, pain is the most frequent complaint. Uterine fibroids during pregnancy cause complications in roughly 2% of cases and include degeneration, preterm labor and birth, preterm premature rupture of membranes (PPROM), and pyomyoma [3, 4].

A pyomyoma, or suppurative leiomyoma, results when a fibroid becomes infected and necrotic. Typically, the fibroid becomes infarcted during pregnancy or in the postpartum period due to hemorrhage or decreased blood flow, leading to necrosis and infection. The infection then spreads from the fibroid either directly to adjacent structures or by lymphatic or hematogenous spread [4–6]. Previously documented infections are polymicrobial with organisms that are present in the lower genital tract which spread in an ascending fashion or are believed to spread directly when there is direct contact with the endometrial cavity [5, 6]. Commonly documented organisms are Staphylococcus aureus, Streptococcus haemolyticus, Proteus, Streptococcus agalactiae, E. coli, Enterococcus faecalis, and Sphingomonas paucimobilis [5]. A rare complication of pyomyoma is a spontaneous uterine rupture, which can lead to worsening sepsis and death. In a majority of cases, the patient will require hysterectomy, and fertility is unable to be preserved. However, there are a few documented cases where conservative management was successful [6, 7]. This is a case report describing the postpartum course of a primipara, complicated by pyomyoma, that was managed successfully with fertility-sparing treatment.

## 2. Case Presentation

**Figure 1:**
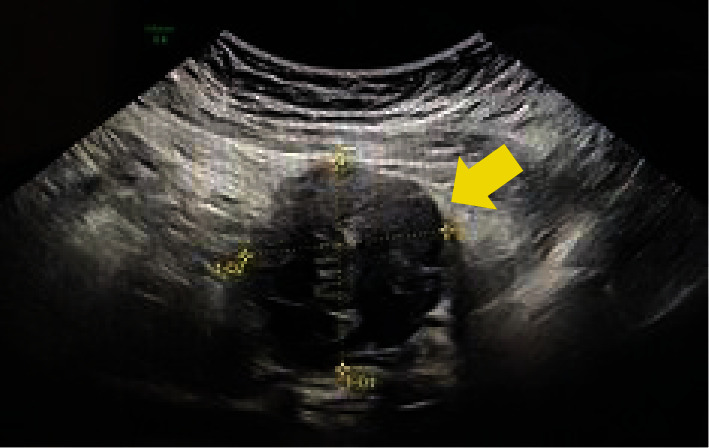
Image of the subserosal fibroid measuring 3.8 × 3.2 × 4.0 cm on the patient's initial ultrasound performed at 10-week gestational age.

The patient was a 31-year-old G2P0111 who presented to her primary obstetrician 7 days after an uncomplicated vaginal delivery with a complaint of lower abdominal pain. Her pregnancy had been complicated by iron deficiency anemia, fibroid uterus (Figure 1), and a history of COVID-19. She reported that the lower abdominal pain was localized to the right lower quadrant, described as stabbing, nonradiating, and exacerbated by movement. A bedside abdominal ultrasound was performed by the physician that was suspicious for a fluid collection in the abdomen. The patient was then sent to the emergency room for further evaluation.

**Figure 2:**
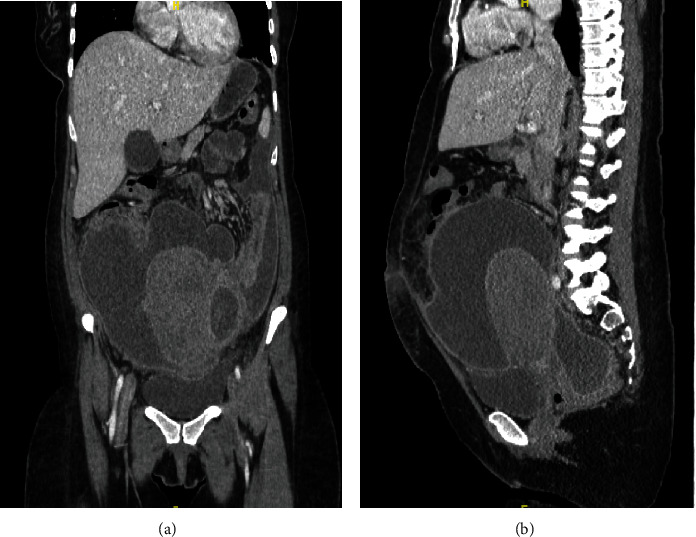
Coronal (a) and sagittal (b) views of the CT abdomen and pelvis with IV contrast performed in the emergency department demonstrating multiple, large loculated fluid-filled collections.

Initial computed tomography (CT) of the abdomen and pelvis was significant for multiple, large loculated fluid-filled collections within the peritoneum measuring 21.4 cm by 21.1 cm by 16.5 cm with a separate fluid collection in the cul-de-sac measuring 7.0 cm by 5.0 cm by 8.9 cm (Figure 2); previously noted fibroid was not visualized. However, at the time of this CT scan, radiology did not have access to the ultrasound images from the OBGYN's office, so they were not aware of the previously documented fibroid uterus. The findings were concerning for phlegmons or early abscesses. Significant laboratory findings included leukocytosis with a WBC count of 17.4 × 10^3^ *μ*L, thrombocytosis with platelets of 703 × 10^3^ *μ*L, and hypokalemia with potassium at 3.0 mmol/L. Due to these collective findings, the patient was admitted for management with intravenous (IV) antibiotics, and interventional radiology (IR) was consulted. The patient was started on IV ampicillin and sulbactam, gentamicin, and clindamycin. A Jackson-Pratt (JP) drain was placed in the right lower abdominal quadrant by IR under CT guidance, with drainage of 900 cc of yellow, purulent fluid. The sample was submitted for culture and cytology. Infectious disease was also consulted, and the IV clindamycin was subsequently discontinued.

On the third hospital day, blood cultures had been negative for approximately 36 hours, and culture of the fluid drained grew Fusobacterium nucleatum. Her leukocytosis reached an apex of 22.7. Infectious disease escalated her antibiotics from gentamicin and ampicillin/sulbactam to piperacillin and tazobactam.

**Figure 3:**
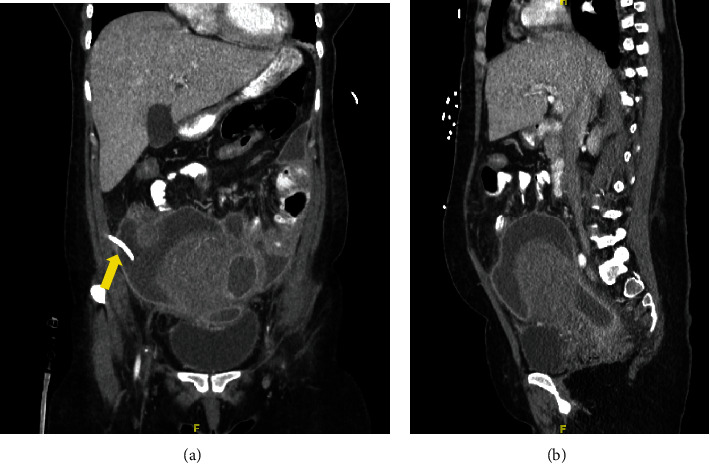
Coronal (a) and sagittal (b) views of the repeat CT abdomen and pelvis with IV and PO contrast performed 3 days after IR drainage and JP drain (arrow) placement still demonstrating multiple, large loculated fluid-filled collections.

During the following 3 days, her WBC count decreased and there was minimal output from the JP drain. Repeat imaging was ordered for evaluation of the abscess prior to removal of the JP drain. Despite improvement in her WBC count and JP drain output, repeat CT imaging of the pelvis and abdomen showed only a small decrease in the fluid collected from the right lower quadrant near the JP drain. Overall, the fluid collection remained large, measuring 17.8 cm by 8.5 cm by 14.2 cm with the fluid collection in the cul-de-sac measuring 6.1 cm by 4.9 cm by 7.5 cm (Figure 3). IR was consulted again for placement of an additional drain. At this time, the gynecology team reviewed her CT imaging with IR and discussed the patient's medical history including uterine fibroid, which IR was not previously aware of. After learning about the fibroid and the location, IR was concerned for possible uterine rupture. Due to this suspicion and minimal improvement, IR recommended surgical exploration.

The patient was consented and underwent a diagnostic laparoscopy that was converted to an exploratory laparotomy. Upon initial visualization of the intra-abdominal cavity, extensive adhesions were noted, and general surgery was consulted intraoperatively. However, despite lysis of adhesions, they were unable to obtain adequate visualization of the pelvis. The decision was made to convert to laparotomy. During lysis of adhesions, multiple small bowel serosal tears were found. A majority of the serosal tears were able to be repaired, but two areas were noted to be too significant for repair. Ultimately, the patient required a small bowel resection. The surgeon also performed drainage and debridement of intra-abdominal/pelvic abscess and fluid.

After a bowel resection and extensive irrigation of the abdominal cavity, the pelvic cavity was able to be inspected. The uterus was localized and was noted to have a 3 × 3 cm defect extending more than 50% into the myometrium in the left anterior fundus. This was the location of the previously described fibroid, which was no longer identifiable. This finding was concerning for spontaneous uterine rupture. The uterine rupture was considered to be the likely source of the abscess formation. The uterine defect was subsequently closed in an interrupted fashion using 1 chromic suture. A sample of the debridement was obtained and sent to pathology for evaluation. After further exploration, no other sources for abscess formation were identified. Two 19 FR Blake drains were placed, one located posterior to the uterus and the other within the left gutter, in close proximity to the uterine repair.

The Blake drains remained in place until POD 4, and she had her diet advanced as tolerated, from clear liquids to a regular diet by the general surgery team. The patient was transitioned to p.o. amoxicillin by infectious disease and was discharged on postop day 5 in stable condition. She was instructed to complete a 5-day course of amoxicillin and to follow up in one week with both the obstetric and general surgery teams. After discharge, her postoperative recovery was uneventful, and she did not require any further hospital admissions or reoperations.

The pathology report of the surgical debridement noted severe acute and chronic inflammation and necrotic tissue with adjacent fibrous/smooth muscle tissue showing changes consistent with infarct. The overall architecture of the fibrous/smooth muscle tissue was consistent with the uterine wall. These findings confirmed our suspicion that the patient experienced a fibroid infarction during the postpartum period, which created a pyomyoma that lead to a spontaneous uterine rupture and the formation of intra-abdominal abscesses.

## 3. Discussion

Pyomyoma is an extremely rare complication of uterine fibroids during pregnancy or the postpartum period. One study reported that only 13 cases of pyomyoma related to pregnancy have been documented since 1945 [7]. While pyomyoma is a rare complication of fibroids, the mortality rate is reported to be as high as 21-30% [8]. A pyomyoma typically presents with fever, leukocytosis, tachycardia, pelvic pain, and characteristic features on imaging studies. On ultrasound, a heterogenous uterine mass with cystic components or air can be seen, as well as an anechoic halo of normal myometrium surrounding the mass [9]. On CT scan, calcifications, free peritoneal air, and fluid with the density of purulent material may be visualized [10]. While imaging can be suggestive of a pyomyoma, it is ultimately a surgical diagnosis. Imaging findings can also be suggestive of spontaneous uterine rupture, including seeing disruption of the wall of the uterus with free intraperitoneal fluid and air [6].

Management of pyomyoma includes IV antibiotics and, in most cases, surgical management. A majority of patients require a hysterectomy and fertility is unable to be preserved. There have been a few documented cases where pyomyoma has been managed more conservatively with antibiotics and CT-guided drainage or myomectomy, in order to preserve fertility [6]. Specifically, Read and Mullins reviewed 41 pyomyoma cases since 1986 and found 49% of patients had a hysterectomy, 34% had a myomectomy, and 17% were treated with IV antibiotics and minor surgery. This case review study included patients who were not postpartum and women who were postmenopausal, but it demonstrated that conservative management was used in a minority of the cases. Our case was initially managed conservatively, similar to other documented cases, with broad-spectrum IV antibiotics and IR drainage of the abscess. Ultimately, our patient required more aggressive management with exploratory laparotomy to drain the intra-abdominal and pelvic abscesses. However, our more aggressive approach allowed us to adequately assess the uterus and discover the spontaneous uterine rupture, which was the source of her abscesses and infection. We were able to repair the defect and this allowed us to preserve her fertility. While we had a favorable outcome, there was a delay in diagnosis of the spontaneous uterine rupture because the radiology department did not have access to the ultrasound performed in the clinic. If IR had been made aware of the fibroid sooner, they likely would have been concerned for a uterine rupture earlier in her hospital admission. She likely would have had surgical management sooner, and this potentially could have prevented the small bowel injuries which lead to resections. Also, this would have decreased the length of her hospital stay. This demonstrates the importance of communication with radiology and any specialist involved in a patient's care, making sure they are aware of a patient's past medical history.

While pyomyomas are extremely rare, they are associated with high morbidity and mortality. Due to their rarity, a high index of suspicion must be present from physicians in order to achieve a correct diagnosis and provide prompt treatment. Findings that should raise suspicion for pyomyoma would be signs of sepsis and pelvic pain in the setting of a fibroid uterus. Ultrasound and CT imaging can help guide the diagnosis, but it is ultimately a surgical diagnosis. Conservative management in order to preserve fertility is reasonable to consider and can be successful, as demonstrated by our case.

## Data Availability

The data supporting this case report can be accessed through http://PubMed.gov.
